# Health outcomes, education, healthcare delivery and quality – 3063. Cost-effectiveness of immunotherapy for children with atopic dermatitis

**DOI:** 10.1186/1939-4551-6-S1-P228

**Published:** 2013-04-23

**Authors:** Vladislava Derkach, Tatiana Slavyanskaya

**Affiliations:** 1 Vladivostok State Medical University, Vladivostok, Russia; 2 Allergology & Immunology, University of Russia, Moscow, Russia

## Background

The study was aimed at evaluating the cost-effectiveness of immunotherapy (IT) for children (Ch) with atopic dermatitis (AD).

## Methods

There were examined 94 Ch aged 3-18 years with AD and duration of disease 1-15 years. Patients underwent the standard general clinical and immunological and allergy survey. The treatment consisted of 2 stages: traditional therapy (TT) for reducing disease complications, and IT. Patients were divided in 3 groups: I group (n=30) received stepped therapy with immune response-modulating agent (IRMA) in course dose of 20mg; II group (n=31) was treated with parenteral accelerated allergen-specific immunotherapy (APAI); III group (n=33) received combination of IT - CIT (IRMA+APAI) on the fast track scheme. Pharmacoeconomic assessments were performed using cost-effectiveness analysis.

## Results

The cost of a pack of IRMA was $7.67. The cost of 1 year APAI was $32.2. After 2 courses of IRMA the TT costs in I group were $189.69±1.08, in II group– $69.14±0.93, and in III group– $32.34. Three years of 2 consecutive courses in I group resulted in mean treatment costs of $569.06; 3 consecutive courses in II group– in mean treatment costs of $104.00, and $97.01 in III group for the same period. In addition, CIT provided long-term remission, reduced number of hospitalization, prevented AD progression, improved skin condition of children with AD, thus normalizing emotional balance. Furthermore there was indirect costs minimization - 2/3 reduction of parents’ sick leaves payments.

## Conclusions

The clinico-economic analysis showed that despite the complexity of APAI, CIT method improves the quality of life of children with AD, provides pharmacotherapy and medical services costs reduction, and it is more cost-effective.

